# Effect of non-surgical periodontal treatment on clinical signs of rheumatoid arthritis

**DOI:** 10.15171/japid.2018.003

**Published:** 2018-06-20

**Authors:** Fazele Atarbashi-Moghadam, Fahimeh Rashidi Maybodi, Ali Dehghan, Alireza Haerian Ardakani

**Affiliations:** ^1^Department of Periodontics, Dental School, Shahid Beheshti University of Medical Sciences, Tehran, Iran; ^2^Department of Periodontics, Faculty of Dentistry, Shahid Sadoughi University of Medical Sciences, Yazd, Iran; ^3^Department of Rheumatology, Shahid Sadoughi University of Medical Sciences, Yazd, Iran; ^4^Department of Orthodontics, Faculty of Dentistry, Shahid Sadoughi University of Medical Sciences, Yazd, Iran

**Keywords:** Chronic periodontitis, rheumatoid arthritis, periodontal treatment, non-surgical

## Abstract

**Background:**

Several studies have shown the association between periodontitis and rheumatoid arthritis (RA) and some similarities in the pathogenesis of these two diseases but clinical trials which have evaluated the effect of periodontal therapy on clinical signs of active RA are scarce, particularly in Iran. The aim of this study was to evaluate the effect of non-surgical periodontal treatment on the improvement of clinical signs of patients with RA.

**Methods:**

Fifty-six patients with active RA and generalized moderate-to-severe chronic periodontitis were selected and divided into case (periodontal treatment) and control (without periodontal treatment) groups. Periodontal parameters and RA disease activity scores (DAS28 index) were compared at 6- and 12-week intervals.

**Results:**

In the case group, a significant reduction in all the periodontal indices was observed 6 weeks after treatment. At the 12-week evaluation, periodontal indices showed a significant reduction in comparison with baseline and a little increase in comparison with 6-week interval. Six weeks after treatment, DAS28 decreased significantly in the case group (P<0.05). DAS28 also decreased at 12-week interval but its reduction was not significant in comparison with 6-week interval. DAS28 in the case group at 12-week interval was significantly less than that in the control group (P<0.05).

**Conclusion:**

Since periodontal care as a part of treatment protocol in RA patients can be effective in improving clinical signs, the recall intervals are better to be 3 months or shorter.

## Introduction

Periodontitis is one of the most common chronic oral diseases characterized by connective tissue destruction and alveolar bone loss.^1^ Rheumatoid arthritis (RA) is an autoimmune chronic destructive disease characterized by the accumulation and persistence of inflammatory infiltrate in the synovial membrane that leads to synovitis and destruction of joints and finally, functional disability. RA afflicts 0.5‒1% of the population worldwide.^1,2^ These two chronic inflammatory diseases have some similarities in their clinical and pathological features.^1,3-5^ Similar immunological response, active tissue destruction, cellular infiltrations and inflammatory mediators (PGE-2, TNF-∝ and IL-1) exist in both diseases.^6,7^ Antibodies against a variety of periopathogens were found in the serum and synovial fluids from patient with RA.^8-10^ Rheumatoid factor (RF) has been detected in the gingival tissue, subgingval plaque, saliva and serum of patients with periodontal diseases. However, the characteristics and precise function of RF in periodontal disease has not yet been recognized.^11,12^

In spite of the vague relationship between these two diseases,^1,13,14^ Pinho et al^13^ suggested that the periodontal treatments are important in inflammation control and avoidance of tooth extraction for patients with RA and periodontitis. Silverste et al^14^ concluded from their systematic review that a decrease in periodontal inflammation contributed to clinical improvements in RA but the available evidence is insufficient and further studies are necessary. In this case‒control study we evaluated the impact of nonsurgical periodontal therapy on RA-related factors.

## Methods

The registration code in Iranian Registry of Clinical Trials for this study was IRCT201202219104N1. This study was performed in accordance with the Declaration of Helsinki and subsequent revisions^15^ and was approved by the Ethics Committee of Shahid Sadoughi University of Medical Sciences. Written informed consents were obtained before participating in the study from the patients admitted into the Clinic of Rheumatology, Shahid Sadoughi Hospital, Yazd, Iran. Fifty-six participants with active RA and moderate-to-severe periodontitis were selected.

### Diagnosis of RA

The diagnosis of active rheumatoid arthritis was based on features defined by the American College of Rheumatology classification criteria for rheumatoid arthritis. The number of tender joints, number of swollen joints and ESR (erythrocyte sedimentation rate) level were recorded. Pain score was also recorded for each individual using visual analog scale (VAS). VAS is a self-reported scale which has 10 scores (0‒10) from no pain to the worst possible pain. The DAS28 index (disease activity score) was calculated by placing these recorded factors in a particular formula in a programmed calculator application.

### Diagnosis of periodontitis

Periodontal status was examined by a periodontist. Patients who had at least 20 teeth and had generalized moderate-to-severe chronic periodontitis were selected. According to the 1999 workshop of periodontology, the generalized form was classified as involvement of more than 30% of sites and moderate form was considered as the presence of sites with 3‒4 mm of attachment loss and severe as the presence of sites with ≥5 mm of attachment loss. The periodontal status was checked by assessing the probing depth (PD) and bleeding on probing (BOP). Oral health conditions in all the participants was measured using O'Leary plaque index (PI).^16^

Exclusion criteria consisted of antibiotic use during a 3-month period before the study, smoking, diabetes, heart disease, history of radiation therapy or chemotherapy, severe xerostomia, pregnancy and lack of informed consent.

### Study design

Patients who refused periodontal treatment were classified as the control group (28 patients) and age- and sex-matched patients who were interested in periodontal treatment served as the case group (28 patients). Two groups were matched based on the severity of periodontitis according to the extent of attachment loss and rheumatoid arthritis according to baseline DAS28 values. Moreover, as far as possible, dosages and types of drugs were similarly distributed in both groups. The pattern of patients' medication remained unchanged until their next visit.

Oral health instructions, including the method of brushing and flossing properly, were given to all the patients. In the case group, scaling and root planing were performed using an ultrasonic device (Woodpecker, China) and the teeth were polished by our collaborating periodontist. Then after 6 and 12 weeks, probing depth, MGI and BOP were measured in both groups by another periodontist. Then DAS28 scores of patients in both groups were again assessed by our rheumatologist and finally comparisons were made between the case and control groups in terms of this index. It should be mentioned that patients in the control group were informed that they could withdraw from the study whenever they wanted and periodontal treatment began for them immediately.

During this study, nine patients were excluded and replaced by nine new patients because of not taking part in the follow-up appointments at 6th and 12^th^ weeks or non-compliance with poor plaque control after periodontal treatment. After collecting and coding, data were analyzed with SPSS 15, using chi-squared test and t-test.

## Results

The case group (periodontal treatment) included 28 patients (6 males and 22 females) aged 32‒60 years (mean age of 45.07±4.93 years) and the control group (without periodontal treatment) included 28 patients (6 males and 22 females) aged 33‒60 years (mean age of 45.17±4.35 years). The two groups were not significantly different in terms of age (P=0.960) and sex (P=1.000).

### Baseline (T1)

The difference between mean PI (PI1) in both groups was not statistically significant (P=0.953). The mean PD (PD1) of the two groups was not significantly different, either (P=0.833). The participants in both groups were not significantly different in terms of baseline BOP (BOP1) (P=0.763). The mean of DAS28 at baseline between the two groups did not differ significantly (P=0.803) (Table 1).

**Table 1:** The means of periodontal parameters in the case and control groups at baseline (T1) and 6 (T2) and 12 weeks (T3) after periodontal treatment

**Periodontal parameters**	**Baseline (T1)**		**After 6 weeks (T2)**		**After 12 weeks (T3)**	
	**Case**	**Control**	**Case**	**Control**	**Case**	**Control**
Plaque index (%)	52.29	52.14	40.54	45.18	42.54	49.82
Probing depth (mm)	5.46	5.50	4.39	5.62	4.78	5.60
Bleeding on probing (%)	33.86	33.46	29.71	34.25	30.43	35.29
DAS28	4.25	4.26	3.56	3.87	3.52	3.71

### Evaluation after 6 weeks (T2)

The PI2 significantly decreased in both groups but the case group exhibited a greater decrease (P<0.001 in both groups). PD2 changes in the control group were not significant, although it showed a slight increase (P=0.304). In the case group, these changes exhibited a statistically and clinically significant decrease (P=0.001). BOP2 exhibited a significant decrease in the case group (P=0.001). However a slight increase was observed in the control group but this difference was not statistically significant (P=0.504). DAS28 in both groups decreased significantly after 6 weeks, which was greater in the case group (P0.001 in both groups) (Table 1).

### Evaluation after 12 weeks (T3)

The mean PI3 increased in both groups compared to PI2, which was not significant in the case group (P=0.299). PI3 was still significantly lower than PI1 in the case group (P=0.0001). In the control group, PI3 was not significantly different from PI1 (P=0.254). PD3 was significantly higher in the case group compared to PD2 (P=0.022) but PD3 was still significantly lower compared to PD1 (P=0.0001). In the control group, changes in PD3 compared to PD1 (P=0.477) and PD2 (P=0919), were not statistically significant. BOP3 in the case group exhibited no significant difference in comparison to BOP2 (P=0.650) and it was still significantly lower than BOP1 (P=0.025). BOP3 in the control group exhibited no significant difference compared to BOP2 (P=0.356) and BOP1 (P=0.148). In both groups, the baseline DAS28 exhibited a significant decrease compared to the 12th week (P=0.0001 in both groups). In the 12th week, DAS28 values in the case group were significantly lower than the control group (P=0.015) (Table 1).

## Discussion

Fifty-six patients with active rheumatoid arthritis and moderate-to-severe periodontitis were selected and divided into two groups. In the beginning, the two groups were matched for age and gender. The difference between the means of age in the case and control groups were not statistically significant (45.07 years and 45.17 years, respectively) (P<0.05). Each group consisted of 22 females (78.6%) and 6 males (21.4%). Higher female-to-male ratio among patients admitted to the clinic was consistent with the epidemiological pattern of RA which shows a higher incidence in women.^2^ Sample size in this study (n=28 per group) was higher as compared to the limited studies with periodontal treatment intervention, for example Ortiz et al^17^ (20 patients in each group), Pinho et al^13^ (15 patients in each group) or Al-Katma et al^18^ studies (17 patients in the treatment group and 12 patients in the control group).

In this study, PI decreased significantly after 6 and 12 weeks. PI3 was slightly greater than PI2. This may showed the importance of follow-up and oral hygiene instruction reinforcement. PI2 in the control group significantly decreased, which was possibly due to the effects of oral hygiene instructions that provoked the desire for oral healthcare in the patients. Furthermore, the consequent awareness of being studied, might have improve the participants’ oral hygiene behaviors. Such positive effect is called “Hawthorne effect" in health studies.^19^ However, PI3 was very close to PI1. The mean PD significantly decreased after periodontal treatment (PD2) in the case group. Mean value of PD3 was significantly higher than PD2 but the difference was not clinically significant. Mean PD changes were not significant in the control group. In the case group of this study, BOP decreased significantly after 6 and 12 weeks of treatment, which might be attributed to periodontal treatment and decreased inflammation. BOP3 slightly increased compared to BOP2, which was not significant. In the control group, BOP changes were not statistically significant.

Both groups showed a significant decrease in DAS28 after 6 weeks, which was maintained after 12 weeks (P=0.0001 in both groups). DAS28 values at 6- and 12-week intervals in the case group were significantly lower than the control group (P=0.015). The medicine dosage and drug charts of the two groups were very similar. Significant decrease in DAS28 of controls could be due to rheumatoid arthritis treatment. The difference between the amounts of decrease in the two groups was significant, and the higher decrease in the case group could be due to the positive effects of periodontal treatments.

Earlier studies on the effect of periodontal treatment on RA improvement had different case and control selection and methods; therefore, the comparison was difficult but all of them concluded that nonsurgical periodontal treatment improved the signs and symptoms of RA. Al-katma et al^18^ studied patients with RA and mild-to-moderate periodontitis (17 patients in the treatment group and 12 patients in the control group) and after 8 weeks of follow-up reported significant decreases in PI, PD, Bop and DAS28. Ortiz et al^17^ studied 40 patients with moderate/severe RA and severe periodontitis (20 patients in each group) and showed that nonsurgical periodontal therapy resulted in significant decreases in PI, PD, BOP and DAS28 in the control group after six weeks. Pinho et al^13^ found significant decreases in PI and PD and non-statistically significant reduction in BOP after 3 and 6 months of follow-up of 15 patients with RA and periodontitis, who received periodontal treatment and 15 patients with RA and periodontitis without periodontal treatment. They reported that DAS28 decreased significantly after three months; however, these changes were not significant after six months.^13^ Erciyas et al^20^ assessed 60 patients with chronic periodontitis (30 with moderately/highly active RA and 30 with low activity RA) 3 months after nonsurgical periodontal treatment. They observed that periodontal treatment might have beneficial effects on reducing low, moderately and highly active RA. However, they did not have a control group without treatment.^20^ In a study by Ribeiro^21^ after 3 months, they compared RF with ESR to evaluate the RA condition. ESR levels were significantly lower in the treated groups. RF was also lower but its reduction was not significant. RF is not regarded as a specific test for rheumatoid arthritis because the test can be positive in 3‒5% of healthy people and this factor is also found in patients with other autoimmune diseases (e.g. Sjögren’s syndrome) and infectious diseases (e.g. hepatitis and tuberculosis).^22^ On the other hand, laboratory tests do not provide any data regarding the number of affected joints or the pain felt by the patient. DAS28 is related to ESR, as an inflammatory index, and also the clinical aspect of the disease. Therefore, in this study it was selected as the activity index of rheumatoid arthritis instead of RF. Two systematic reviews in this issue showed that nonsurgical periodontal therapy had beneficial effects on clinical and laboratory tests of RA patients but the body of evidence was small.^5,14^

## Conclusion

All the findings of this study indicated that for acquiring a durable effect of phase I treatment the patients should be recalled and treated at <3-month intervals. Studies have shown re-colonization of bacteria in periodontal pockets three months after treatment.^23^ The positive effects of phase one of periodontal therapy in patients with rheumatoid arthritis continued for three months after treatment. Thus, periodontal treatment, besides regular follow-ups, can be helpful in improving rheumatoid arthritis symptoms.

## Competing interests

The authors declare no conflict of interests.

## Ethics approval

The registration code in the Iranian Registry of Clinical Trials for this study was IRCT201202219104N1. This study was performed in accordance with the Declaration of Helsinki and subsequent revisions and was approved by the Ethics Committee of Shahid Sadoughi University of Medical Sciences under the code 50352.
